# Quality of life among type 2 diabetes mellitus patients at Kamuzu Central Hospital in Lilongwe, Malawi: A mixed-methods study

**DOI:** 10.1371/journal.pgph.0002367

**Published:** 2023-10-09

**Authors:** Alinafe Chisalunda, Wingston Felix Ng’ambi, Nesto Salia Tarimo, Ndaziona Peter Kwanjo Banda, Adamson Sinjani Muula, Johnstone Kumwenda, Alinane Linda Nyondo-Mipando

**Affiliations:** 1 Department of Physiotherapy, Mangochi District Hospital, Mangochi, Malawi; 2 Department of Health Systems and Policy, School of Global and Public Health, Kamuzu University of Health Sciences, Lilongwe, Malawi; 3 Department of Rehabilitation Sciences, Kamuzu University of Health Sciences, Blantyre, Malawi; 4 Department of Medicine, Kamuzu University of Health Sciences, Blantyre, Malawi; 5 Department of Community and Environmental Health, Kamuzu University of Health Sciences, Blantyre, Malawi; 6 Johns Hopkins Project, Blantyre, Malawi; PLOS: Public Library of Science, UNITED STATES

## Abstract

Type II diabetes mellitus (T2DM) significantly impacts quality of life (QoL) yet data among these patients in Malawi are lacking. This study was conducted to assess QoL among patients with T2DM. A mixed-method cross-section study was conducted at Kamuzu Central Hospital (KCH), Lilongwe, Malawi. Data collection was done using a modified diabetes quality of life (MDQoL)-17 questionnaire for quantitative data while in-depth interviews and diary methods were used for qualitative data. Demographic data were summarized using descriptive statistics and inferential statistics using t-tests and ANOVA. Thematic analysis was utilized for qualitative data. A sample of 339 participants (mean age 50.3±15.5) was recruited. Overall, the mean QoL score was moderate (mean QoL 63.91±19.54). Those on health insurance had better QoL (QoL 76.71, C.I. 69.22–84.19, p-value 0.005) compared to those without health insurance. Furthermore, the absence of comorbidities was associated with having better QoL (QoL 71.18, C.I. 66.69–75.67, p-value < 0.0001). Qualitatively, T2DM was associated with patients’ health status, increased stress levels, and loss of independence. There were QoL-promoting factors among T2DM patients such as diabetes health talks, having a supportive family, and following hospital advice. Inhibiting factors include drug shortages, societal perceptions, a sedentary lifestyle, stress, and despising hospital advice. Overall QoL in patients with T2DM receiving treatment at KCH is moderate. QoL of patients with T2DM is influenced by interrelated factors which require multidisciplinary team care to optimize the QoL among these patients. Health workers need to adopt a holistic approach when treating patients with T2DM, such as managing comorbidities and including assessment of QoL, behavioral change measures like physical exercises, and a healthy diet.

## Introduction

Diabetes mellitus is among the top 10 leading causes of death globally [1]. In the African region in 2021, the prevalence of diabetes was estimated at 4.5% among people aged 20–79 years [2]. In Malawi, an epidemiological transition has occurred with the prevalence of diabetes increasing from less than 1% in the 1960s to 5.6% in 2009 [2, 3]. As of 2019, the prevalence was 4.5% among adults aged between 20 and 79 years [4]. Currently, diabetes services in Malawi, are provided in all district hospitals though the services are challenged by a lack of resources and skilled health personnel [5, 6]. There is a small proportion of primary health facilities with adequate resources for the screening and treatment of diabetes in Malawi [5, 6].

In working to address challenges in the management of diabetes and other non-communicable diseases (NCDs), the Ministry of Health established the National non-communicable Disease and Injuries (NDCI) poverty Commission in 2015 with support from the Lancet NCDI poverty commission [7]. This commission identified 38 priority NCDI conditions and proposed a set of 54 proven, cost-effective interventions to address these conditions [7].

Diabetes mellitus(DM) is one of the NCDs that significantly impacts and reduces QoL among patients [8]. The use of QoL as a measurable outcome in health has recently gained attention as healthcare has shifted from a disease-focused biomedical model [9] to a more holistic, well-being-focused biopsychosocial model [10, 11]. However, QoL is often ignored in the overall assessment of health outcomes [12].

Despite the significance of QoL studies among diabetes patients, there is limited evidence of such studies in Malawi. Quality of life assessment in diabetes patients is important because it ensures the individualization of patients’ treatment according to their complaints and different diabetes complications [13]. This study, therefore, assessed the quality of life among type II diabetes mellitus patients at KCH in Lilongwe, Malawi.

## Materials and methods

### Study design and setting

A convergent mixed-method study employing a cross-section design for quantitative data and a phenomenological approach for qualitative data was conducted at the outpatient diabetes clinic at KCH. This is the second largest tertiary public hospital located in the capital city of Lilongwe district in Malawi that provides referral services from 5 district hospitals and serves a population of 4 million [14]. The hospital’s diabetes clinic is one of the largest in the country. It runs two times per week and serves an average of 80 patients per day. Furthermore, Lilongwe district has the highest population in the country (1,276,000 as of 2022) [15] and it is for these reasons that the findings of the study can be generalized for the country.

### Study participants and recruitment

For the quantitative part, three hundred thirty-nine (339) patients with T2DM for a duration not less than 1 year and receiving care at an outpatient diabetes clinic at KCH, were included in the study. These patients were aged 18 years and above. The duration of the presence of diabetes was ascertained by asking the patient verbally to give information about when they were diagnosed with T2DM. Sample size calculation was arrived at using the formula; N = Z^2^xp(1-p)/d^2^. Where Z is the standard normal variate at 5% type 1 error which is 1.96. Whereas p is the overall QoL score of 51.50% that was found in a similar study done at Mizan Tepi University Teaching Hospital in Ethiopia [16]. This study was chosen based on the assumption that Malawi and Ethiopia have almost similar demographic characteristics and health care challenges. In addition, no similar study has been done in Malawi prior hence using the Ethiopian study. *d* is the absolute error which in this study is estimated at 5%.

For the qualitative part, a purposive sample of eighteen patients with T2DM (6 for in-depth interviews and 12 for the diary method) was drawn from the quantitative sample of 339 participants. The diary method (S1 Text) is essential because it allows events to be recorded in their natural setting and captures data from participants as they live through certain experiences [17]. Six guardians for patients with T2DM who were consistently staying with the patients for more than 2 weeks were also included for in-depth interviews in the qualitative part. In total, 24 participants were included in the qualitative part.

The sample size for qualitative data was based on the assumption that saturation for the phenomenological approach is reached with 5 to 25 participants [18]. In addition, 6 to 12 interviews are enough to achieve the desired research objective [18].

### Data collection and analysis

Data collection was done from 6^th^ April to 18^th^ June 2021. The MDQoL-17 tool (S2 Text) was used to collect quantitative data. The tool has seven domains which include physical functioning, role limitations due to physical health problems, role limitations due to personal or emotional problems, emotional well-being, social functioning, energy and fatigue, and general health perceptions [12]. This tool is appropriate because it is a diabetes-specific QoL measuring tool and covers all domains of QoL [19] which covered the objectives of the study. An interview guide (S3 Text) and a digital voice recorder were used to collect qualitative data, field notes were also taken to enrich the data [20]. Furthermore, patients were provided with diaries (S1 Text) to write their daily personal experiences, at least three times a week for one month.

MDQoL-17 questionnaire and in-depth interviews were piloted on other diabetes patients to ensure that they were relevant and applicable. To ensure the reliability and validity of quantitative data, an existing questionnaire (MDQoL-17) was used which was developed and validated in India by Prajapati et al. in 2017 [12]

After data collection, STATA software was used for quantitative analysis. Demographic characteristics were summarized using descriptive statistics. QoL scores were calculated using the MDQoL-17 questionnaire where question scores were calculated as percentages with 0 being the lowest score and 100 being the highest score according to Prajapati et al. 2017 (Table 1) [12]. After scoring the questions, QoL was categorized into three parts. A QoL score <50 was poor, a score between 50 and 70 was moderate and a score of more than 70 signified better QoL [12].

**Table 1:** Response category and scores of MDQoL-17 questions [12].

Item number	Response category and scores
1,2,7,13	1 = 100, 2 = 75, 3 = 50, 4 = 25, 5 = 0
3	1 = 0, 2 = 25, 3 = 50, 4 = 75, 5 = 100
4,5,6	1 = 0, 2 = 50, 3 = 100
8,9,10,11,12,14,15,16	1 = 0, 2 = 20, 3 = 40, 4 = 60, 5 = 80, 6 = 100
17	1 = 100, 2 = 80, 3 = 60, 4 = 40, 5 = 20, 6 = 0

For comparison of QoL scores and demographics as well as comorbidities, an unpaired t-test was applied for the means of two groups and ANOVA for three or more groups. These are parametric tests and a p-value of less than 0.05 was considered significant.

For qualitative data, thematic analysis was used. Firstly, audio data were transcribed into written form [21]. Secondly, the researchers familiarized themselves with the data through repeated reading of the transcripts [21]. The generation of codes was the third step, the point at which researchers identified and highlighted raw data or information that had a common pattern [21]. The codes were developed through both inductive and deductive approaches [22]. The fourth step required the researchers to aggregate all codes with similar meanings into different groups that are called themes [21]. After that, researchers reviewed and refined the devised themes to ensure that the codes were grouped correctly. This was followed by naming of the themes [21] and finally, the researchers wrote all the themes into a report.

### Ethical consideration

Ethical approval was obtained from the College of Medicine Research and Ethics Committee (COMREC) reference number P.09.20.3122. The Director of KCH granted institutional support to conduct the study at the facility. Participation was voluntary and written informed consent was obtained from the participants. They were informed of their right to withdraw from the study at any point without affecting their access to medical services.

## Results

Since this was a mixed methods study with both quantitative and qualitative sections, the results are presented separately starting with quantitative and then qualitative results.

### Quantitative results

#### Characteristics of persons with type II diabetes mellitus

There were 339 patients with T2DM enrolled in this study with the majority being females (64.3%) and the mean age was 50.3±15.5 years. Forty percent were overweight (BMI; 25–29.9kg/m^2^) with a mean BMI of 26.6 ±5.5 kg/m^2^ (Table 2).

**Table 2:** QoL and characteristics of persons with type II diabetes mellitus.

Patient Characteristics (n = 339)	N	%	Mean QoL (95% CI)	P-value
**Total**	**339**	**100.0**		
**Sex**				0.63
Males	121	35.7	64.59(61.16–68.03)	
Females	218	64.3	63.54(60.90–66.17)	
**Agegroups (years)**				0.05
<40	92	27.1	61.64(57.52–65.76)	
41–65	187	55.2	66.23(63.49–68.97)	
>65	60	17.7	60.18(55.22–65.15)	
**BMI (kg/m** ^**2**^ **)**				0.02
<18.5	21	6.2	53.86(45.70–62.01)	
18.5–24.9	104	30.7	63.64(60.02–67.27)	
25–29.9	135	39.8	62.97(59.73–66.21)	
30+	79	23.3	68.56(64.05–73.07)	
**Treatment duration (years)**				0.56
<5	137	40.4	64.74(61.47–68.02)	
06–010	114	33.6	62.29(58.68–65.92)	
>10	88	26.0	64.72(60.61–68.82)	
**Education level**				0.005
None	39	11.5	58.21(51.81–64.60)	
Primary	134	39.5	62.70(59.43–65.97)	
Secondary	131	38.6	64.21(60.82–67.61)	
Tertiary	35	10.3	73.8(68.56–79.04)	
**Marital status**				0.87
Divorced	13	3.8	63.77(52.63–74.91)	
Married	245	72.7	64.24(61.79–66.69)	
Single	30	8.9	61.07(53.75–68.39)	
Widowed	51	15.0	64.08(58.72–69.44)	
**Medical aid**				0.005
No	322	95.0	63.24(61.10–65.38)	
yes	17	5.0	76.71(69.22–84.19)	
**Alcohol drinking**				0.49
No	329	97.1	63.79(61.67–65.90)	
Yes	10	3.0	68.1(54.59–81.61)	

Forty percent of the participants had primary school education. Out of 339 participants, 73% were married, 5% were on health insurance, 98% reported no alcohol drinking and none of the participants reported a history of smoking (Table 2).

#### Assessment of quality of life among persons with type II diabetes mellitus

The Median for QoL was 63.33(56–71). A QoL score <50 was poor, a score between 50 and 70 was moderate and a score of more than 70 signified better QoL (Table 3). Forty-one percent of patients had QoL between 70–100 and twenty-four percent had a QoL of less than 50 (Table 3).

**Table 3:** Assessment of quality of life among persons with type II diabetes mellitus.

Quality of Life categories	N	%	Median QoL(IQR)
**TOTAL**	**339**	**100**	**63.33(56–71)**
<50	82	24.2	
50–70	117	34.5	
70–100	140	41.7	

#### Quality of life varied by demographic and behavioral characteristics

Patients who had a tertiary education had better QoL than those who had secondary, primary and no education (QoL score 73.8, 95%CI 68.56–79.04 vs QoL score 64.21, 95%CI 60.82–67.61, QoL score 62.70, 95%CI 59.43–65.97, QoL score 58.21, 95%CI 51.81–64.60 respectively, p-value 0.005). There was an increasing trend in the QoL by education level. Similarly, patients who had health insurance had a better QoL than those who did not have health insurance (QoL score76.71, 95%CI 69.22–84.19 vs QoL score 63.24, 95%CI 61.10–65.3, p-value 0.005). Furthermore, patients who were obese (BMI >30kg/m^2^) had a better QoL compared to those who were overweight, had normal weight and underweight (QoL score 68.56, 95%CI 64.05–73.07 vs QoL score 62.97, 95%CI 59.73–66.21, QoL score 63.64, 95%CI 60.02–67.27, QoL score 53.86, 95%CI 45.70–62.01 respectively, p-value 0.02) (Table 4).

**Table 4:** Quality of life varied by demographic and behavioral characteristics.

Patient Characteristics (n = 339)	N	%	Mean QoL (95% CI)	P-value
**Total**	**339**	**100.0**		
**BMI (kg/m** ^**2**^ **)**				0.02
<18.5	21	6.2	53.86(45.70–62.01)	
18.5–24.9	104	30.7	63.64(60.02–67.27)	
25–29.9	135	39.8	62.97(59.73–66.21)	
30+	79	23.3	68.56(64.05–73.07)	
**Education level**				0.005
None	39	11.5	58.21(51.81–64.60)	
Primary	134	39.5	62.70(59.43–65.97)	
Secondary	131	38.6	64.21(60.82–67.61)	
Tertiary	35	10.3	73.8(68.56–79.04)	
**Medical aid**				0.005
No	322	95.0	63.24(61.10–65.38)	
Yes	17	5.0	76.71(69.22–84.19)	

#### Quality of life varied by comorbidities and complications

QoL was further assessed based on the presence and absence of comorbidities as well as complications (Table 5). In comparison with patients with comorbidities or complications, those without comorbidities or complications had a better QoL (QoL score 71.18, 95%CI 66.69–75.67 vs QoL score 61.2, 95%CI 58.97–63.45, p-value <0.0001). Specifically, patients without musculoskeletal diseases had a statistically significantly better QoL than those with musculoskeletal diseases. (QoL score 66.01, 95%CI 63.48–68.54 vs QoL score 66.01, 95%CI 63.48–68.54, p-value 0.002).

**Table 5:** Assessment of quality of life based on comorbidities and complications.

Comorbidities/complications	N	%	Mean qol (95%CI)	P-value
**Total**	**339**	**100**		
**Comorbidities/complications**				<0.0001
Absent	92	27.1	71.18(66.69–75.67)	
Present	247	72.9	61.21(58.97–63.45)	
**Cardiovascular diseases**				0.07
No	283	83.5	64.77(62.49–67.06)	
Yes	56	16.5	59.57(54.54–64.60)	
**Musculoskeletal diseases**				0.002
No	238	70.2	66.01(63.48–68.54)	
Yes	101	29.8	58.98(55.48–62.48)	
**Urologic diseases**				0.79
No	335	98.8	63.88(61.78–69.99)	
Yes	4	1.2	66.5(52.67–80.33)	
**Reproductive system diseases**				0.32
No	329	97.4	64.10(61.97–66.22)	
Yes	10	3.0	57.9(46.85–68.95)	
**Endocrine diseases**				1
No	330	97.4	63.92(61.80–66.03)	
Yes	9	2.7	63.89(49.56–78.22)	
**Neurology diseases**				0.13
No	267	78.8	64.75(62.34–67.17)	
Yes	72	21.2	60.81(56.84–64.78)	
**Ophthalmic diseases**				0.26
No	267	78.8	64.54(62.16–66.91)	
Yes	72	21.2	61.61(57.26–65.96)	

#### Common comorbidities and complications with type II diabetes mellitus patients

Of the 73% that had comorbidities and complications, the most common comorbidities/complications reported by patients were cardiovascular diseases (16.5%), musculoskeletal diseases (29.8%), neurologic conditions (21.2%), and ophthalmic conditions (21.2%). A few patients reported having urologic diseases (2.7%), reproductive (3%), and endocrine system conditions (1.2%) (Table 5).

#### Sub-groups of people who were more likely to be affected

**Fig 1:**
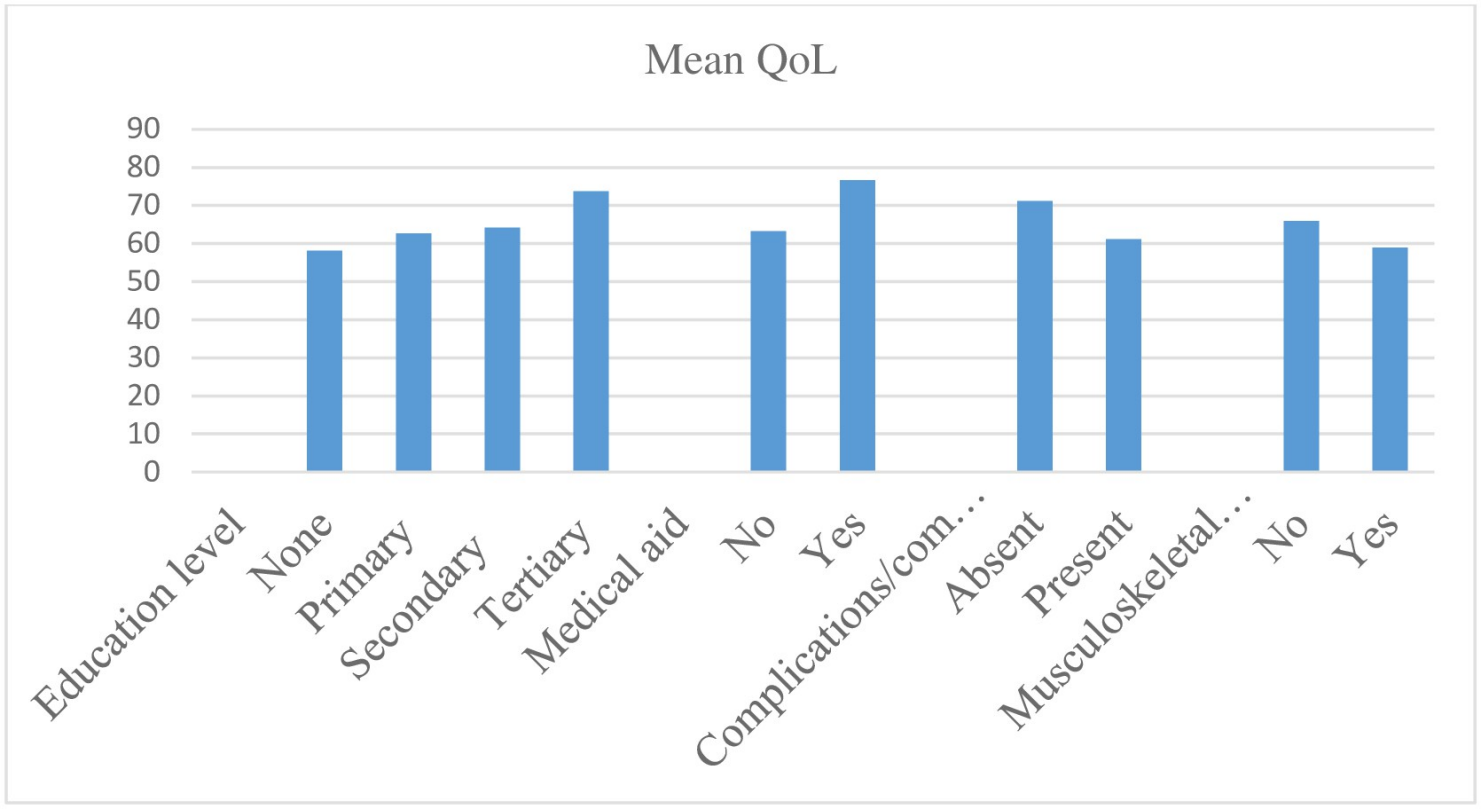
Sub-groups of people who were more likely to be affected measured by their QoL scores.

Sub-groups of people who were more likely to be affected were those with no education compared to those who had primary, secondary and tertiary education (QoL score 58.21, 95%CI 51.81–64.60 vs QoL score 62.70, 95%CI 59.43–65.97, QoL score 64.21, 95%CI 60.82–67.61, QoL score 73.8, 95%CI 68.56–79.04 respectively, p-value 0.005). Similarly, patients who did not have health insurance were more likely to be affected compared to those who had health insurance (QoL score 63.24, 95%CI 61.10–65.3 vs QoL score76.71, 95%CI 69.22–84.19, p-value 0.005). In comparison with patients with comorbidities or complications, those with comorbidities or complications were more likely to be affected compared to those who did not have comorbidities or complications. (QoL score 61.2, 95%CI 58.97–63.45 vs QoL score 71.18, 95%CI 66.69–75.67, p-value <0.0001). Specifically, patients with musculoskeletal diseases were more likely to be affected than those without musculoskeletal diseases. (QoL score 66.01, 95%CI 63.48–68.54 vs QoL score 66.01, 95%CI 63.48–68.54, p-value 0.002) “Fig 1”.

#### Domains of life that were most negatively affected

**Fig 2:**
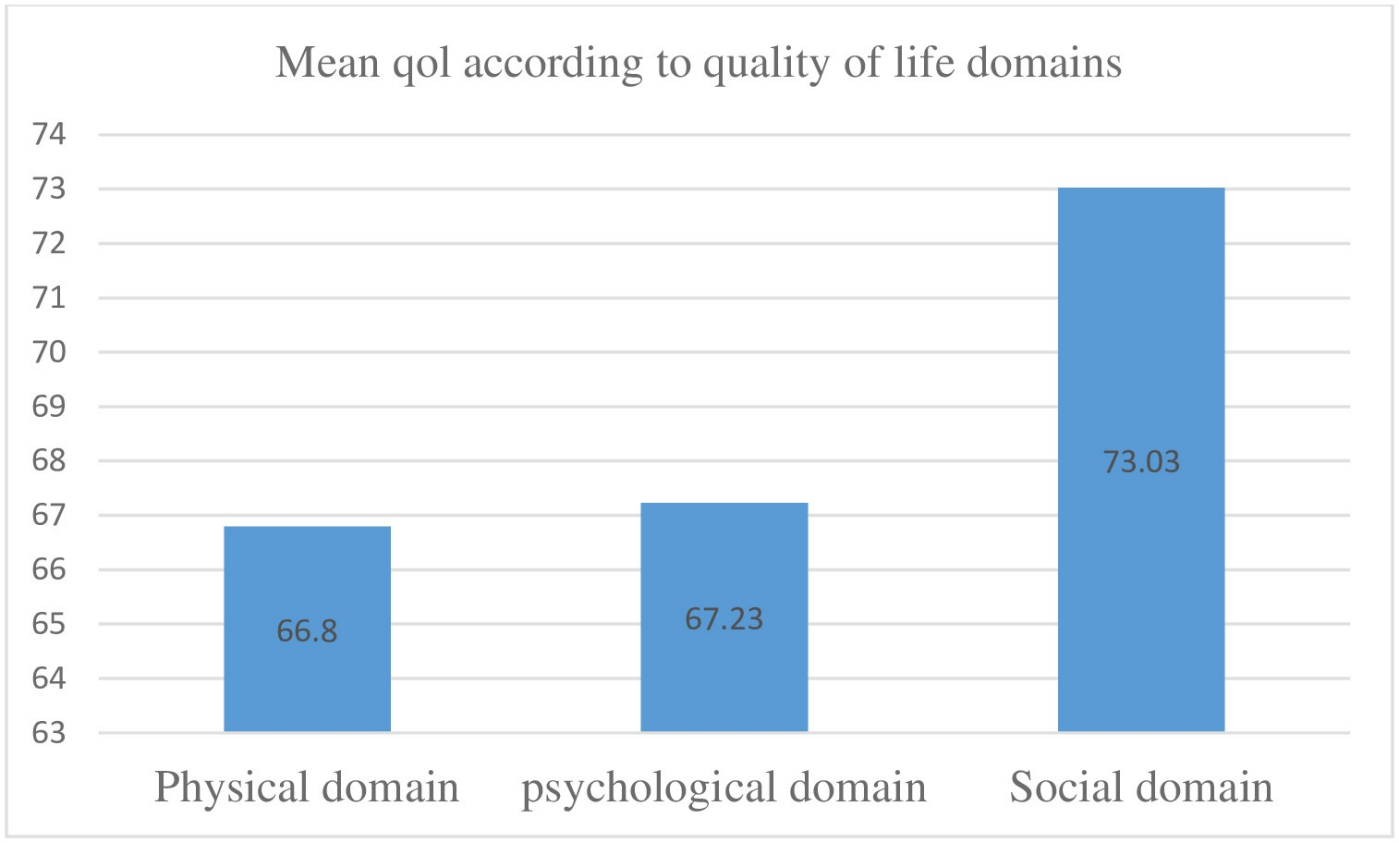
Quality of life according to quality of life domains.

The MDQOL-17 questionnaire was divided into three domains of life; physical, psychological and social domains. The most negatively affected domain was the physical domain (QoL score 66.80, 95%CI 64.22–69.37) compared to psychological and social domains (QoL score 67.23, 95%CI 64.46–70.01 and QoL score 73.03, 95%CI 70.55–75.51 respectively) “Fig 2”.

### Qualitative results

#### Characteristics of interviewees and diarists

A total of 12 participants participated in the in-depth interviews: their ages ranged from 25 to 72 years of whom seven were females. Six of the interviewees were T2DM patients, of whom three were female. Six of the interviewees were guardians of T2DM patients and four of them were females. Out of the twelve diaries that were distributed, seven diarists were males. In the end, we managed to collect eight diaries of which five were from male.

From the qualitative results, four themes emerged namely; definition of quality of life, the impact of diabetes on patients, promoters of quality of life among type 2 diabetes mellitus patients and inhibitors of quality of life among type 2 diabetes mellitus patients.

### Theme 1: Definition of quality of life

#### Absence of disease

The participants referred to the absence of disease as not getting sick often and having a body that is resilient to illness.

“According to me the way I see quality life… is the one who is not falling sick often. Because when you become sick frequently, you cannot work to bring wealth to your family, and you can’t manage to work to have enough food for your family. So quality life let’s say it is someone who does not get sick frequently, that’s the one who has quality life, not just riches… no”.

(69 years old male, guardian interviewee)

#### Independence

Quality of life also emerged as an act of independence. The participants felt that one needs to manage taking care of oneself, work satisfactorily and travel without any problems.

“Quality life means living without getting sick quite often, working satisfactorily, and also walking/travelling without any problem”.

(35 years old male patient interviewee)

### Theme 2: Impact of diabetes on patients

The participants explained that diabetes has a lot of impacts on patients because of its complexity and chronic nature. These impacts were categorized into impact on health status and lifestyle.

#### Impact on health status

Some participants reported having body weakness frequently. They also reported that diabetes results in different problems like eye-sight, wounds that are difficult to heal which may end up in the loss of limbs through surgery, sudden death, a burning sensation, lack of sexual desire, and numbness, especially in the legs and feet. The patients reported that due to all these problems, they become stressed because they feel that their lives are unstable. They also stated that sometimes stress is caused by loss of independence and failure to take care of their families.

“I hear some people saying that a diabetes patient dies suddenly when their sugar drops extremely. And also, when you are diagnosed with diabetes when you are young, you have never been married, and I heard that you lack sexual desire. And also, when you have a wound or a sore, it is difficult to get it healed and takes a long time. For example, if you get injured on your leg or toe/finger, that part might be removed. As a result, I am stressed a lot. Furthermore, I heard that diabetes may destroy the eyes”.

(Diarist female patient)

Some family members also observed the impact diabetes has on their patients. They reported that diabetes patients feel weak most of the time; they have various problems like poor and loss of eyesight, and also they feel stressed because of their disabilities as well as loss of independence.

“When he was diagnosed with diabetes in 2016 his life was not very healthy. He used to be sick always to the extent of being amputated. So you can understand that after amputation, there was nothing he could do, he doesn’t do anything… but he has a lot of thoughts about how he has failed to provide for his family. So that’s what I see affects him; that as a father, he is supposed to provide for his family, but he doesn’t”.

(25 years old female guardian interviewee)

#### Lifestyle changes

In addition to the above challenges, participants pointed out that diabetes demands multiple changes in someone’s life for example loss of independence, they rely on someone to help them with activities of daily living as well as failing to earn a living.

“Everything has to be done for her… for example cooking for her. She can’t manage to cook what she wants and we may have to cook for her. Maybe washing for her, giving her water to bath. Everything. She has just reached a level whereby it’s like we are taking care of a baby now…”

(49 years old female guardian)

In addition, the participants stated that they are restricted from eating a variety of foods, and as a result they feel stressed as they are always searching for their recommended foods. This also restricts them from travelling freely as they are not sure whether they can find the right food as they travel. The participants further reported that food restrictions prevent them from participating in social gatherings.

“…Sometimes, I may be stressed as diabetes demands that I eat frequently. Sometimes I might feel hungry when I am in a group of people, and I am required to do something according to how I feel… sometimes, for example, when I am supposed to go to a funeral, I don’t go thinking that something bad might happen while I am there. Like how will I explain to people that I am hungry, or maybe for me to leave my friends and tell them that I am going home temporarily to eat. They may say; “why is she acting like she is the only one having this disease?”

(49 years old female patient interviewee)

### Theme 3: Factors that promote quality of life among diabetes patients

The factors associated with promoting QoL among T2DM patients are categorized under health system, family, or societal and individual factors.

#### Health system factors (diabetes health talks)

Participants reported that health education delivered at the facilities help them lead a healthy life if they follow protocols taught during the health talks. These health education sessions include pieces of advice on taking medication properly, the recommended diet, an allowable amount to consume, and avoiding a sedentary lifestyle.

“…But I believe that we can attain a good life if we follow the advice we are taught time and again. A while ago, there was a nurse who used to come before the start of each diabetes clinic to advise us. It was really good but these days she is nowhere to be seen. But if she is no longer here, can’t there be another nurse to encourage and help us reduce our stress so that we can have a good and long life if we can manage to take good care of ourselves?”

(Diarist male patient)

#### Family/societal factors (supportive family, good relationships)

Having a supportive family and good relationships promote the QoL of diabetes patients. The support that relatives render include encouragement to follow the advice received from the hospital, for example, taking medication properly and following a recommended diet. Participants further reported that good relationships help them with psychological support.

“My relatives are good people and they are the only ones who understand my diabetes problem. They encourage me to eat the right diet as well as remind me when to take medicine. They always want to see me happy and stress-free”.

(Diarist female patient)

In agreement with the participants, one guardian reported that supportive families and relationships are good for patients.

“Because when we remind him to come to the hospital, there are also protocols concerning what kind of food he is supposed to eat and also being active most of the time so that his body should not be weak”.

(36 years old man, guardian interviewee)

#### Individual factors

Some of the participants reported that avoiding stress and accepting their condition are key elements in life. They further indicated that when they accept, it is more likely that they follow hospital advice, for example, eating the right food and leading an active lifestyle.

“Most importantly, it is good not to be worried, no… accept the situation. And also when you accept, be settled so that you should not get sick quite often… because if you are not accepting, you are always worried as a result you may die faster”.

(47 years old man patient interviewee)

### Theme 4: Factors that inhibit the quality of life among diabetes patients

Some of the participants in the in-depth interviews explained that there are factors that contribute to reduction of QoL among T2DM patients in addition to the presence of the disease itself. These factors are categorized under the following; health system, family or societal factors, and individual factors.

#### Health system factors (drug shortages)

The participants complained that sometimes they are faced with a shortage of drugs at the hospital which require them to buy from private pharmacies. This becomes a challenge as they report that these drugs are expensive. As such, they become stressed over their ability to access medicines.

“Sometimes we come to the hospital and we are told diabetes medicines are out of stock, and we are forced to go and buy them at pharmacies. Maybe the government should look at that so that we can live in a diabetes-free world”.

(25 years old woman guardian interviewee)

#### Family/Societal factors

Some patients reported that they are labeled as selfish if they refuse to eat a certain type of food at communal gatherings, for example, at a funeral or a wedding. Some people may think that diabetics are nagging unnecessarily when they report getting hungry frequently, consequently limiting their travel and participation in social gatherings.

“This disease doesn’t allow us to eat good, soft, oily, and sweet food. This results in us being painted as selfish because most of our friends do not understand this disease. We are recommended to eat m’gaiwa nsima (a staple food in Malawi) so it is hard for family and friends to accept and assist us accordingly”.

(Diarist male patient)

#### Individual factors

Participants highlighted stress as one of the factors that reduces QoL among diabetes clients. Stress may arise from the presence of diabetes itself and its complex nature. In addition, some patients may despise hospital advice and ignore the prescribed food, medicine, and physical exercises. All of these may put them at risk of getting sick often hence, enhancing instability in their lives and in their relationships.

“…. anxiety disturbs because just the fact that you are worried, disturbs your body. This means that the body doesn’t function properly. Because getting worried and the nature of diabetes do not go together”.

(35 years old man patient interviewee)

“… That’s why if you just eat those fatty foods, for example, at a funeral you had those fatty foods, you just find that the body is not normal. It’s not that you are sick or you have general body pains. Then you realize that: Oh I have made a mistake…”

(69 years old woman patient interviewee)

Guardians corroborated what the participants reiterated about the importance of accepting their condition and adhering to advice as a key to leading a better life. Furthermore, one of the guardians indicated that it is possible to live in a diabetes-free world if diabetes management and preventive measures are followed.

“The way I see it, I think diabetes can be prevented. And I have heard other people recovered from it properly. It just requires a person to follow what the doctor says… so if he can follow instructions given at the hospital, we can live in a diabetes-free world”.

(25 years old woman guardian interviewee)

## Discussion

In this study, QoL was defined as the absence of disease and having an independent life. Factors that promote QoL among T2DM patients are diabetes health talks, having a supportive family, accepting one’s condition positively, and following hospital advice such as doing physical exercises and following a prescribed diet. Factors that inhibit QoL among T2DM patients include drug shortages, societal perceptions, sedentary lifestyles, stress, and despising hospital advice. Demographic and behavioral characteristics and comorbidities or complications, determine the quality of life. T2DM impacts patients’ lives in several ways like food restrictions, loss of independence, changes in lifestyle, and increased stress levels.

### Definition of quality of life and impact of type II diabetes mellitus

Our findings that QoL means absence of disease in an individual as well as an individual’s ability to lead an independent life, are similar to the World Health Organization’s definition that QoL is an individual’s perception of their position in life in the context of the culture and value systems in which they live, and concerning their goals, expectations, standards, and concerns [23]. Previous studies found that T2DM can adversely affect virtually all aspects of a patient’s life [24]. It often leads to a deterioration in the patient’s physical and psychological well-being, a change in their lifestyle and its adaptation to the illness, as well as changes in professional, social and personal values which in turn affect the patient’s QoL [24]. These findings were in agreement with this study, which also found similar ways in which T2DM impacts patients’ lives. In relation to other studies, managing diet and weight is one of the challenges impacting T2DM patients’ daily life [25]. In addition, T2DM patients have problems with self-confidence, and the ability to take on life’s challenges, and they are faced with high levels of stress associated with diabetes management [25]. Literature has further documented that diabetes reduces productivity while working due to increased absenteeism and many working limitations, which result in productivity losses for employers as well as significantly decreasing the probability of subsequent employment [26].

### Determinants of quality of life among type II diabetes mellitus patients

Our findings that many of the study patients had a better QoL are in contrast to findings from a Brazilian study that found that many of the participants had moderate QoL of 50–70 [12].

Although our results suggest that people with medical aid had better QoL, our results are limited because of the small proportion that had medical aid. The majority of participants in this study were not on any health insurance which is consistent with insurance coverage in Malawi [27]. Going forward, Malawi could adopt the Namibian Government’s approach which has one-third mandatory social health insurance funds and about two-thirds voluntary private plans [28] because this has the potential of optimizing QoL. In addition, our results that suggests that medical insurance improves QoL should be interpreted with caution because only 5% of participants had health insurance which makes the estimate imprecise. Future studies should include more people on medical insurance to test this impression further.

Having tertiary education was significantly related to having better QoL (QoL score 73.8, 95%CI 68.56–79.04, p-value 0.005). This is in agreement with a previous study that stated the importance of education for psychological health and well-being [29]. Evidence states that higher education typically leads to occupations that involve less health risk and provide greater financial capacity to purchase better nutrition and health care which are directly linked to health [29]. In addition, higher education has a positive impact on emotional well-being since individuals acquire self-esteem and problem-solving skills [29]. This is a call for the government and various stakeholders to promote education that improves health [30]. Despite obesity being the leading risk factor for T2DM (Clinical risk categories for BMI: normal weight 18.5–24.9 kg/m^2^, overweight 25–29.9 kg/m^2^, and obesity ≥30 kg/m^2^ are associated with a stepwise increase in diabetes risk) [31], in this study, being obese was significantly related to having better QoL (QoL score 68.56, 95%CI 64.05–73.07, p-value 0.02). On the contrary, previous studies have documented that QoL decreases with increasing BMI/obesity (as measured by SF-36v2 scores: normal weight 18.5–24.9 kg/m^2^; score 51.7, overweight 25–29.9 kg/m^2^; score 50.7, and obesity ≥30 kg/m^2^; score 47.8) [32]. This is seen as a call for a similar study to be done with a larger sample size and in more than one study setting.

### Factors that promote quality of life among type II diabetes mellitus patients

Our study showed that health education is key for diabetic clients to lead an optimal life because it raises awareness of various aspects of diabetes mellitus which is essential for the prevention, management, and control of the disease [33, 34]. These educational programs help people assess their risks of diabetes, motivate them to seek proper treatment, and inspire them to take charge of their disease [33, 34].

Increasing the number of facilities offering diabetes services is another strategy that would contribute to the QoL of diabetes patients because it will curb the long distances that patients have to cover to access health services [5, 35, 36]. This can be learned from other African countries that are actively instigating programs to improve the care of patients with T2DM starting with improved diagnosis [37]. Efforts to adequately equip primary health facilities to manage NCDs including diabetes need to be fast-tracked [35].

A supportive family and good relationships also enable diabetes patients to attain better QoL which is consistent with findings from a previous study that showed that family members are key sources of both instrumental and emotional support [38]. This support includes helping patients complete specific tasks, such as making an appointment with health care providers or helping with insulin injections, and emotional support, such as providing comfort and encouragement when patients face distress or frustration over the long course of their diabetes care [38]. These characteristics have been linked to superior health outcomes, including a healthier diet, increased physical activity, and lower rates of mortality across various medical conditions [39]. However, psychological support for diabetes patients is inadequate, resulting in poor QoL and reduced general well-being [40]. Recognizing the influence family can have, Pamungkas et. al. highlighted the need for diabetes care guidelines to include the provision of diabetes education to family members or incorporate family support as part of the patient’s diabetes care plan [38]. Hence, mental health specialists need to empower diabetes patients’ guardians with skills to support diabetes patients psychologically.

Acceptance of the disease leads to adherence to the prescribed treatment, the recommended diet, physical exercises, and living positively [41, 42]. All of these enhance the normalization of their blood sugar and reduce sickness frequency, hence attaining better QoL [41]. Regular physical exercise has proven to be effective in improving glycaemia by lowering insulin resistance and promoting insulin secretion [43]. It also reduces the risk of cardiovascular disease and obesity, the main risk factors for T2DM, in patients with T2DM [43] and they have been classified as key components of the therapeutic approach for T2D individuals [44].

The study had several limitations. Firstly, a cross-section design was used quantitatively, which limited the analysis of causal relationships. But the use of a mixed-method study, different data collection methods, and tools helped to increase the richness of the data and study validity. There is a need to conduct a similar study in more than one setting to better understand what health systems are doing differently that can optimize the QoL of diabetes patients. In similar studies, it is also important to ascertain glycemic control since it has a bearing on the incidence of diabetes complications and therefore QoL. Since data collection was done during the COVID-19 pandemic, some patients refused to participate because they thought they would be vaccinated against COVID-19. Some patients reported that they have been participating in several studies but have never received any results hence refusing to participate. Some diaries were not collected for several reasons. For instance, this was a maize harvesting season and some of these diarists were busy in their fields. Some diarists’ mobile phones were unreachable when called to arrange for diary collection and some did not pick up their mobile phones during all attempts.

## Conclusion

Overall QoL in T2DM patients is moderate (median 63.33(56–71)) since a QoL score <50 was poor, a score between 50–70 was moderate and a score of more than 70 signified better QoL using the MDQoL-17 tool. The QoL of diabetic patients is influenced by interrelated factors, so efforts to optimize QoL should be multidisciplinary. Inclusion of guardians in the care of diabetic patients is critical to adherence to recommended treatment and lifestyle, which are key to promoting QoL. Health workers need to adopt a holistic approach when treating patients with type II diabetes mellitus and include assessment of QoL, behavioral change measures like physical exercises, and a healthy diet. The government and various stakeholders need to promote education that improves health status.

## Supporting information

S1 Text Diarist instructions. (DOCX)

S2 Text Modified diabetes quality of life questionnaire (MDQOL-17). (DOCX)

S3 Text A. In-depth interview guide for patients. B. Guardian in-depth interview guide for qualitative data.
(ZIP)
