# Supplementary material for: Quality of life among type 2 diabetes mellitus patients at Kamuzu Central Hospital in Lilongwe, Malawi: A mixed-methods study
Source: PLOS Glob Public Health. 2023 Oct 9;3(10):e0002367. doi: 10.1371/journal.pgph.0002367 (PMC10561856; doi:10.1371/journal.pgph.0002367)
Supplement: S1 Text — (DOCX) [file pgph.0002367.s001.docx]

**DIARIST INSTRUCTIONS**

**Title: Quality of life among patients with type II diabetes mellitus at Kamuzu central hospital in Lilongwe, Malawi**

ID/name of Diarist _________________

Location of the Diarist______________

Phone number of Diarist__________________________________

Period of entry: First Day of entry__________Last day of entry__________

Language: English or Chichewa

**Guidance on Diary Entries**

- Write daily if possible but at least 3 times a week for one month over anything about the following:
- General body physical functioning
- Any problems if any concerning physical health
- Any personal/emotional problems
- Any problems with emotional well-being
- When you feel fatigue
- Any problems if any with social participation
- Any other general health problems

For example:

*I am about to cook for my family but I feel very weak. Now I remember that I did not take my diabetes medication.*

Or

*I am supposed to go to my friend’s daughter’s wedding but I have remembered that there is no proper rest room at that venue. Mmmmm, I think I should not go because I visit the rest room frequently*

At any time you are writing in the diary, indicate date and time.
