# Supplementary material for: Quality of life among type 2 diabetes mellitus patients at Kamuzu Central Hospital in Lilongwe, Malawi: A mixed-methods study
Source: PLOS Glob Public Health. 2023 Oct 9;3(10):e0002367. doi: 10.1371/journal.pgph.0002367 (PMC10561856; doi:10.1371/journal.pgph.0002367)
Supplement: S2 Text — (DOCX) [file pgph.0002367.s002.docx]

**MODIFIED DIABETES QUALITY OF LIFE QUESTIONNAIRE (MDQoL-17)**

**Title: Quality of life among patients with type II diabetes mellitus at Kamuzu central hospital in Lilongwe, Malawi**

1. How is your overall health after being diagnosed as diabetic?

- Excellent
- Very good
- Good
- Fair
- Poor

1. Diabetes has worsened your quality of life?

- Not at all
- Slightly
- Moderately
- Quite a bit
- Extremely

1. You get sick quite often compared to others?

- Definitely true
- Mostly true
- Don’t Know
- Mostly false
- Definitely false

1. Walking for Normal daily chores (like going to work or market)?

- Yes Limited a Lot
- Yes Limited a Little
- No Not limited at All

1. Climbing several flights of stairs?

- Yes limited a lot
- Yes limited a little
- No not limited at all

1. Climbing one flight of stairs?

- Yes limited a lot
- Yes limited a little
- No not limited at all

1. Diabetes is affecting your Work life?

- Not at all
- Slightly
- Moderately
- Quite a bit
- Extremely

1. Do you feel downhearted or depressed?

- All of the time
- Most of the time
- A good bit of time
- Some of time
- A little of time
- None of the time

1. Is Diabetes affecting your peace of mind?

- All of the time
- Most of the time
- A good bit of time
- Some of time
- A little of time
- None of the time

1. Do you feel scared when you think about living with diabetes?

- All of time
- Most of the time
- A good bit of time
- Some time
- A little of time
- None of the time

1. Whether diabetes made you feel lost since it restricts the food items you like?

- All of time
- Most of the time
- A good bit of time
- Some time
- A little of time
- None of the time

1. Is diabetes making you lose your confidence in your abilities?

- All of the time
- Most of the time
- A good bit of time
- Some of time
- A little of time
- None of the time

1. Is diabetes is affecting your Family life?

- Not at all
- Slightly
- Moderately
- Quite a bit
- Extremely

1. Do you feel embarrassed managing your Diabetes in public (like taking tablets/injecting the medicine)?

- All of the time
- Most of the time
- A good bit of time
- Some of the time
- A little of time
- None of the time

15. Whether Diabetes is a hindrance when you are planning for any travel

- All of the time
- Most of the time
- A good bit of time
- Some of the time
- A little of time
- None of the time

16. Is Diabetes bringing up economic burden to you?

- All of the time
- Most of the time
- A good bit of time
- Some of the time
- A little of time
- None of the time

17. Do you feel Energetic?

- All of the time
- Most of the time
- A good bit of time
- Some of time
- A little of time
- None of the time
