# Supplementary material for: Quality of life among type 2 diabetes mellitus patients at Kamuzu Central Hospital in Lilongwe, Malawi: A mixed-methods study
Source: PLOS Glob Public Health. 2023 Oct 9;3(10):e0002367. doi: 10.1371/journal.pgph.0002367 (PMC10561856; doi:10.1371/journal.pgph.0002367)
Supplement: S3 Text — A. In-depth interview guide for patients. B. Guardian in-depth interview guide for qualitative data. (ZIP) [file pgph.0002367.s003.zip › S3A_Text.docx]

**S3A: IN-DEPTH INTERVIEW GUIDE FOR PATIENTS**

**Title: Quality of life among patients with type II diabetes mellitus at Kamuzu central hospital in Lilongwe, Malawi**

Date …………………………………………………………………………………………….

Participant’s code …………………….............................................................................

Researcher/Assistant …………………………………………………………………………

**PART A: DEMOGRAPHIC DETAILS**

**Instructions**

Please tick √ in the box to the right of the answer you choose.

1. Age: ……………………………………………………………………………
2. Gender: Male Female
3. BMI: ……………………………………………………………………………..
4. Diabetes mellitus duration: 1-5 years 6-10 years 11-15 years

16-20 years 21-25 years 26-30 years

31-35 years 36-40 years

Above 40 years

1. Presence of complications/Comorbidities: yes No
2. Education level …………………………………………………..

**PART B: QUESTIONS**

**Instructions**

I will be asking for clarification during the interview, to get a better understanding of your experiences

1. What do you understand by the term quality of life?
   1. Why? (at each response)
2. Does diabetes affect quality of life?
   1. In what ways?
   2. Why(for each response)
   3. For example(s)
   4. Why? (for each response)
3. Describe to me any physical challenges you may have experienced arising from type II diabetes mellitus?
   1. Probe on the physical aspects not mentioned
   2. How do you cope with the each challenge?
4. What about any psychological challenges?
   1. Probe on other psychological aspects not mentioned
   2. How do you cope with each challenge?
5. Any social challenges?
   1. Probe on other social aspects not mentioned
   2. How do you cope with each challenge?
6. What do you think are the contributing factors to these problems?
   1. Probe on health system factors
   2. Probe on family and community factors
   3. Probe on individual factors
7. In your opinion, what can be done to prevent the above problems and hence improve quality of life?
   1. Probe on health system factors
   2. Probe on family and community factors
   3. Probe on individual factors
