# Supplementary material for: Quality of life among type 2 diabetes mellitus patients at Kamuzu Central Hospital in Lilongwe, Malawi: A mixed-methods study
Source: PLOS Glob Public Health. 2023 Oct 9;3(10):e0002367. doi: 10.1371/journal.pgph.0002367 (PMC10561856; doi:10.1371/journal.pgph.0002367)
Supplement: S3 Text — A. In-depth interview guide for patients. B. Guardian in-depth interview guide for qualitative data. (ZIP) [file pgph.0002367.s003.zip › S3B_Text.docx]

**GUARDIAN IN-DEPTH INTERVIEW GUIDE FOR QUALITATIVE DATA**

**Title: Quality of life among patients with type II diabetes mellitus at Kamuzu central hospital in Lilongwe, Malawi**

Date …………………………………………………………………………………………….

Participant’s code …………………….............................................................................

Participant’s phone number ………………………………………………………………….

Researcher/Assistant …………………………………………………………………………

**PART A: DEMOGRAPHIC DETAILS**

1. Age________________________
2. Gender: Male Female
3. Education level …………………………………………………………

**PART B: QUESTIONS**

**Instructions**

Please answer all these questions, I will be asking for clarification during the interview, to get a better understanding of your experiences

1. What do you understand by the term quality of life?
2. After staying with your relation from the time they were diagnosed with diabetes, in what ways do you think type II diabetes mellitus affect quality of life?
3. Describe to me if there are any physical challenges arising from type II diabetes mellitus that your relation might have faced?
4. Any psychological challenges arising from type II diabetes mellitus that your relation might have faced?
5. In what ways does your relation’s condition affect you and your entire family?
6. In your opinion, what can be done to prevent the above problems and hence improve quality of life?
